# MicroRNA-binding site polymorphisms in hematological malignancies

**DOI:** 10.1186/s13045-014-0083-3

**Published:** 2014-11-25

**Authors:** Agnieszka Dzikiewicz-Krawczyk

**Affiliations:** Institute of Human Genetics, Polish Academy of Sciences, Strzeszyńska 32, Poznań, 60-479 Poland

**Keywords:** microRNA, miRNA-binding site polymorphism, Leukemia, Lymphoma, Myeloma, Risk, Prognosis

## Abstract

Dysregulation of microRNA networks has been implicated in hematological malignancies. One of the reasons for disturbed miRNA-mediated regulation are polymorphisms in miRNA-binding sites (miRSNPs), which alter the strength of miRNA interaction with target transcripts. In the recent years the first findings of miRSNPs associated with risk and prognosis in hematological malignancies have been reported. From the studies described in this review miRSNPs not only emerge as novel markers of risk and prognosis but can also lead to better understanding of the role of miRNAs in regulating gene expression in health and disease.

## Introduction

The non-protein coding parts of the genome have been recognized as key players in the regulation of gene expression. Among various classes of non-coding RNAs, the best known are microRNAs (miRNA), small (~22 nucleotide) RNA molecules. MiRNAs bind to complementary target sequences usually located in the 3’ untranslated region (3’UTR) of mRNAs and act predominantly by negatively regulating gene expression [1]. According to the miRBase database, a catalog of miRNA sequences in animals, plants and viruses, so far over 2500 mature human miRNAs have been characterized [2]. Since miRNAs are predicted to regulate over 60% of human protein-coding genes [3], it is not surprising that they have been shown to regulate a plethora of biological processes, including cell proliferation, apoptosis, differentiation and metabolism [4]. Several lines of evidence have demonstrated that miRNAs are required for normal hematopoiesis, while miRNA dysregulation has been associated with the pathogenesis of hematological malignancies [5,6].

The crucial step in the process of miRNA-mediated regulation of gene expression is recognition of the target transcript by miRNA. In animals this recognition is based on pairing of the nucleotides 2-7 of miRNA (so called ‘seed’ region) with complementary sequences in the mRNA, which are usually located in the 3’UTR, but can also be present in the 5’UTR or in the coding region. In addition, pairing of nucleotides 13-16 of the miRNA enhances the miRNA-mRNA interaction and the efficiency of miRNA binding depends also on target site accessibility and secondary structure of the miRNA-target duplex [7]. The requirement for a strict Watson-Crick pairing between the ‘seed’ region of miRNA and its target transcript implies that genetic variability in the 3’UTR can affect miRNA binding by destroying existing sites or creating new miRNA-mRNA interactions. Computational analysis identified hundreds of single nucleotide polymorphisms (SNPs) located within predicted and experimentally verified miRNA-binding sites or potentially creating novel sites for miRNA binding. Moreover, there is evidence for a strong negative selection on SNPs in miRNA-binding sites (miRSNPs) as compared to the entire 3’UTR sequence [8-10], which supports the functional significance of those sites. In the recent years several studies have shown association of miRSNPs with cancer and other diseases [11,12]. In the field of hematological malignancies the first evidence for a disease-associated miRSNP came in 2012 [13] and since then six other studies have been published. Here I will systematically review the so far presented evidence for the relevance of miRSNPs for leukemias, lymphomas and myeloma.

### *KRT81* and *XPO5* in lymphomas and myeloma

The prognostic value of six SNPs located in either genes encoding proteins involved in the miRNA biogenesis or in miRNA-binding sites located in myeloma-related genes was evaluated in 137 patients with multiple myeloma who underwent autologous stem cell transplantation [13]. Association with survival was observed for two SNPs: CC genotype of rs3660 in *KRT81* conferred longer overall survival (OS), while patients with CC/AC genotype of rs11077 in *XPO5* had significantly longer OS and progression-free survival (PFS) (Table 1). KRT81 belongs to a family of hair keratins which are involved in maintaining cell integrity and regulating cell motility and growth in epithelial cells, and have been described as prognostic markers in epithelial tumors [14]. The C allele of rs3660 in *KRT81* reduced significantly the protein level in luciferase reporter assay in one of two myeloma cell lines (RPMI-8226). Also in healthy lymphocytes a significant reduction of KRT81 protein, but not mRNA, level was observed. Furthermore, a significant decrease of the proliferation rate of RPMI-8226 cells was observed upon silencing of the *KRT81* gene, which indicates the possible mechanism underlying the better prognosis in myeloma patients carrying the CC genotype of *KRT81*_rs3660. However, although the authors indicate that according to bioinformatics prediction the C allele destroys binding sites for several miRNAs, results of their experiments indicate otherwise – that in myeloma cell lines and healthy lymphocytes this SNP enhances binding of some miRNAs, which results in decreased protein levels. Since levels of specific miRNAs were not controlled in the luciferase assay, it is not clear whether the observed effect is a consequence of altered miRNA binding, and if so – of which specific miRNA. rs3660 in *KRT81* was also studied in Hodgkin lymphoma (HL) [15]. Here, the GG genotype was found more frequently in patients (30.9%) than in the European population (18.3%, data from the HapMap project) but this should be validated in a control group ethnically matched with patients. Further analysis showed that although rs3660 had no influence on survival of HL patients, the GG genotype is an independent risk factor for treatment-related neurological toxicity. In non-Hodgkin lymphoma (NHL), in turn, distribution of rs3660 genotypes did not differ between patients and control group but carriers of CC and CG genotypes had significantly longer OS compared to the GG genotype [16]. The studies in lymphomas did not attempt to verify whether rs3660 in *KRT81* affects miRNA binding and what are the functional consequences of the polymorphism.

**Table 1:** **Polymorphisms in miRNA-binding sites in hematological malignancies**

**Gene**	**SNP ID**	**Variants**	**Predicted to affect binding of**	**Disease**	**Cases**	**Controls**	**Parameter**	**OR (95% CI), p value**	**Effect on miRNA binding and gene/protein expression**	**Ref.**
*KRT81*	rs3660	G > C	miR-17, miR-20a/b, miR-93, miR-106a/b, miR-519d	MM	137		OS	p = 0.037	Effect on protein expression confirmed in gene reporter assay and *in vivo*	[13]
				HL	139		Treatment-related toxicity	6.65 (1.33-33.26), p = 0.021	Not examined	[15]
				NHL	210		OS	p = 0.012	Not examined	[16]
*XPO5*	rs11077	A > C	miR-4763-5p	MM	137		OS	p = 0.012	Effect on protein expression confirmed in gene reporter assay	[13]
							PFS	p = 0.013		
				HL	127		Treatment-related toxicity	0.49 (0.006-0.376), p = 0.004	Not examined	[15]
							DFS	p = 0.039		
							OS	p = 0.033		
*TP53*	187 novel SNVs		majority of SNVs located in miRNA-binding sites	DLBCL	491		OS	p = 0.019	Effect of three SNVs on miR-125b binding and protein expression confirmed in gene reporter assay	[18]
							PFS	p = 0.018		
							in patients with mutated p53 CDS			
*C14orf101*	rs4901706	G > A		NHL	359		OS	p = 0.015	Effect on protein expression confirmed in gene reporter assay	[19]
				NHL	210	233	Risk	NS	Not examined	[16]
							OS	NS		
*NPM1*	rs34351976	T > -	miR-337-5p, miR-887	AML	93		OS	p = 0.016	Effect on miR-337-5p binding and protein expression confirmed in gene reporter assay, effect on mRNA levels confirmed *in vivo*	[21]
							RFS	p = 0.007		
*ETV6*	rs1576313	T > C	miR-34c-5p, miR-449b-5p	Childhood ALL	101	471	Risk	1.9 (1.16-3.11), p = 0.0107	Effect on miR-34c-5p and miR-449b-5p binding and protein expression confirmed in gene reporter assay	[22]
*TLX1*	rs2742038	C > T	miR-492	Childhood ALL	101	471	Risk	3.97 (1.43-11.02), p = 0.0081	No effect on miR-492 binding and protein expression observed in gene reporter assay	[22]
*PML*	rs9479	G > A	miR-510-5p, miR-589-3p	Childhood ALL	101	471	Risk	0.55 (0.36-0.85), p = 0.0079	Effect on miR-510-5p binding and protein expression confirmed in gene reporter assay; no effect observed for miR-589-3p	[22]
				AML	87	471	Risk	0.6 (0.38-0.97), p = 0.0372		
*ARHGAP26*	rs187729	T > C	miR-18a-3p	CML	140	471	Risk	1.63 (1.07-2.47), p = 0.0213	Effect on miR-18a-3p binding and protein expression confirmed in gene reporter assay	[22]
*IRF8*	rs10514611	C > T	miR-330-3p	CML	140	471	Risk	2.4 (1.12-5.15), p = 0.0246	No effect on miR-330-3p binding and protein expression observed in gene reporter assay	[22]

ALL, acute lymphoblastic leukemia; AML, acute myeloid leukemia; CDS, coding sequence; CML, chronic myeloid leukemia; DFS, disease-free survival; DLBCL, diffuse large B-cell lymphoma; HL, Hodgkin lymphoma; MM, multiple myeloma; NHL, non-Hodgkin lymphoma; NS, not significant; OR (95% CI), odds ratio (95% confidence interval); OS, overall survival; PFS, progression-free survival; RFS, relapse-free survival.

The second polymorphism found to affect survival of patients with multiple myeloma was rs11077 in *XPO5*. This miRSNP, apart from its potential impact on expression of XPO5, can also affect the whole miRNA-ome of the cell as *XPO5* encodes for exportin-5, which is required for the export of precursor miRNAs from nucleus to cytoplasm where they are subject to further steps of maturation [17]. Bioinformatics analysis indicated that the C allele of *XPO5*_rs11077 creates a new binding site for miR-4763-5p. Indeed, luciferase reporter assay showed a significant reduction in protein levels in two myeloma cell lines. Also, in healthy lymphocytes the CC genotype was associated with slightly decreased XPO5 protein levels, but the difference did not reach statistical significance [13]. However, it remains to be established whether miR-4763-5p or another miRNA is responsible for the observed effect. Also, the mechanism by which reduced XPO5 levels could contribute to a better prognosis in multiple myeloma needs to be elucidated. rs11077 in *XPO5* was also studied in Hodgkin lymphoma [15]. Here, the AC genotype was associated with longer overall and disease-free survival (DFS), as well as with lower rate of bleomycin-associated pulmonary toxicity. Better performance of heterozygous patients seems somewhat unusual and probably a replication in a larger cohort of patients could indicate which allele is associated with a better prognosis. This study did not provide any further clues for the functional significance of *XPO5*_rs11077.

### *TP53* and diffuse large B-cell lymphoma

Analysis of the sequence of a known tumor suppressor *TP53* in a cohort of 491 diffuse large B-cell lymphoma (DLBCL) patients revealed a widespread variation in the *TP53* 3’UTR. A total of 187 novel single nucleotide variants (nSNV) and 6 known SNPs were identified. Interestingly, impact of these nSNVs on survival depended on the mutation status in the *TP53* coding sequence (CDS): in patients with the wild type *TP53* CDS nSNVs conferred better 5-year OS and PFS, while in patients with mutated CDS nSNVs were associated with poorer OS and PFS. Bioinformatics analysis showed that majority of the nSNVs were located in validated or putative miRNA-binding sites. Furthermore, it was shown *in vitro* that 3 nSNVs disrupting the binding site for miR-125b increase p53 protein levels (Table 1). However, no correlation was observed between the presence of nSNVs and expression of p53 in tumor samples, regardless of the mutational status of the *TP53* CDS. This could be due to the fact that all nSNVs were analyzed collectively, while some of them may have positive and some negative effect on the p53 expression [18]. This study highlights the relevance of the interplay between miRSNPs and mutations in the coding sequence of a gene. Since the predicted effect of the majority of nSNVs was disruption of miRNA-binding sites, elevated levels of wild type tumor-suppressive p53 would be beneficial, while increased expression of mutated, oncogenic p53 would be unfavourable.

### *C14orf101/TMEM260* in non-Hodgkin lymphoma

Four polymorphisms chosen from a larger set of previously reported miRSNPs [9] were examined for association with cancer risk and prognosis in non-Hodgkin lymphoma. None of them was identified as a risk factor but genotyping was performed on a small sample (96 patients and 90 controls) and should be replicated in a larger cohort. rs4901706 in *C14orf101* was associated with overall survival: the AA genotype was identified as an independent predictor of longer survival in a group of 359 NHL patients. Luciferase reporter assay in the HeLa cell line showed that the G allele significantly reduced protein levels (Table 1) [19]. However, the study provided no indication as to binding of which miRNA might be affected nor the potential functional significance of altered expression of *C14orf101.* Since nothing is known about the functions of *C14orf101,* other than it encodes for a transmembrane protein, it is hard to speculate how altered levels of the protein may affect the prognosis of NHL patients. Interestingly, a team from the same university also studied rs4901706 in a group of 233 controls and 210 NHL patients who were treated at the same hospital and during the same period, and they did not report any association of the SNP with NHL risk or prognosis [16]. This discrepancy highlights the need for studying the functional relevance of miRSNPs to corroborate the findings from association studies.

### *NPM1* in acute myeloid leukemia

Significance of a polymorphic nucleotide T deletion (rs34351976) in the 3’UTR of *NPM1* was studied in acute myeloid leukemia (AML). *NPM1* encodes for nucleophosmin, a molecular chaperone shuffling between nucleus and cytoplasm. Mutations in *NPM1* are found in about one-third of adult AML patients but the exact mechanism by which the mutant NPM1 protein contributes to leukemogenesis remains unclear [20]. Although no difference in the frequency of delT polymorphism was observed between AML patients and healthy controls, Cheng et al. showed that overall and relapse-free survival was significantly worse in patients carrying the homozygous delT. Bioinformatics analysis combined with luciferase reporter assay demonstrated that the delT polymorphism creates an illegitimate binding site for miR-337-5p, which results in decreased protein levels. This finding was confirmed in patient samples: levels of *NPM1* mRNA were significantly lower in patients homozygous for the delT polymorphism than in the non-homozygotes (Table 1). The mechanism by which reduced levels of nucleophosmin resulting from the homozygous delT polymorphism influence outcome of AML patients remains to be elucidated [21].

### 5 miRSNPs in lymphoblastic and myeloid leukemias

Bioinformatics analysis of SNPs in the 3’UTRs of 137 leukemia-associated genes revealed 111 putative miRSNPs. Based on the concordance of at least two algorithms used in the analysis and the expression of predicted miRNAs in blood cells or leukemias and lymphomas, the authors selected 10 miRSNPs for genotyping in patients with childhood acute lymphoblastic leukemia (ALL), adult chronic (CML) and acute myeloid leukemia (AML), and healthy controls. The study showed that homozygous polymorphic genotypes of *ETV6*_rs1573613 and *TLX1*_rs2742038 were associated with increased ALL risk, while carriers of the variant allele of *PML*_rs9479 were at lower risk of ALL and AML. Homozygous polymorphic genotypes of *ARHGAP26*_rs187729 and *IRF8*_rs10514611 conferred increased risk of CML. Furthermore, additive effect of risk genotypes was demonstrated in ALL and CML. Risk increased with each additional risk genotype carried by a patient and surpassed the sum of individual ORs, reaching 13.91 (4.38-44.11) for carriers of three or more risk genotypes in ALL and 4.9 (1.27-18.85) for carriers of two risk genotypes in CML. Effect of significant miRSNPs on miRNA binding was evaluated by luciferase reporter assay and confirmed for three miRSNPs: variant alleles of *ARHGAP26*_rs187729, *ETV6*_rs1573613 and *PML*_rs9479 were shown to affect binding of miR-18a-3p, miR-34c-5p/miR-449b-5p and miR-510-5p, respectively. *TLX1*_rs2742038 and *IRF8*_ rs10514611 were predicted to enhance binding of miR-492 and miR-330-3p, respectively, but this was not confirmed experimentally (Table 1) [22]. The study demonstrated the relevance of polymorphisms in miRNA-binding sites for various types of leukemia, however, the findings should be validated in larger patient cohorts and the effect of miRSNPs on the expression of genes and proteins should be verified *in vivo* in patients samples. Also the mechanisms by which the miRSNPs modulate leukemia risk still needs to be elucidated, especially that the effect of miRSNPs in *ETV6* and *PML* on miRNA binding was not in line with their role as tumor suppressors. This suggests that more complicated miRNA regulatory networks might be affected, according to the concept of ‘competing endogenous RNAs’ (ceRNA) [23].

## Conclusions

More evidence is accumulating for the involvement of deregulated miRNA networks in cancer development. Polymorphisms in miRNA-binding sites of target genes may disrupt miRNA-mediated regulation of gene expression by not only affecting the levels and function of the given protein, but can also interfere with expression of other genes targeted by the same miRNA.

**Figure 1:**
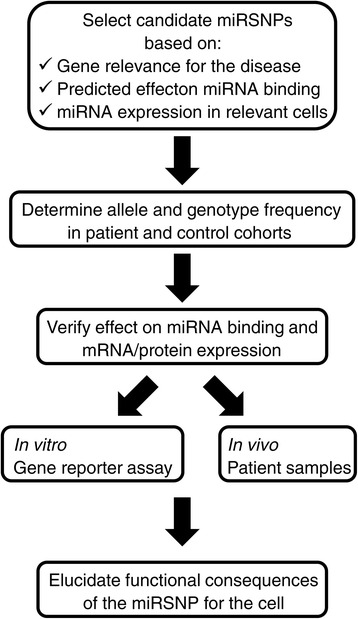
**A plan for a comprehensive study of miRNA-binding site polymorphisms in disease.**

So far only a few studies demonstrated the relevance of miRNA target site polymorphisms for hematological malignancies, but this number will doubtless grow in the next years. The studies presented in this review explored the significance of miRSNPs for the risk or prognosis of blood cancers to a different degree: some did not go beyond reporting a significant association, while others pursued the functional consequences of the miRSNPs. Based on the work described in this review, it is evident that a comprehensive study of miRSNPs in disease should include several carefully planned and performed stages (Figure 1). The first step is the selection of candidate miRSNPs. Thorough scrutiny at this stage will increase the probability of finding miRSNPs of functional importance and facilitate interpretation of the results. Preferably, candidate SNPs should be searched for in genes with proven relevance for the disease in question, which will already give hint for their functional significance. Several tools are available for the prediction of the impact of SNPs on miRNA binding, e.g. miRanda [24], PITA [25], SNPinfo [26] and PolymiRTS [27], and since they use different algorithms for miRNA target prediction, it is advisable to select miRSNPs with the highest support from the available tools. The set of thus selected miRSNPs should be further narrowed down to those predicted to affect binding of miRNAs that are expressed in the relevant cell type, using databases integrating data on miRNA expression across different tissues and cell types, such as mimiRNA [28] or the non-coding RNA body map [29]. Allele and genotype frequencies of the selected miRSNPs are then assessed in appropriate patient and control groups to identify miRSNPs associated with disease risk or prognosis. For statistically significant miRSNPs their impact on binding of miRNAs predicted by the *in silico* analysis should be verified, ideally both *in vitro* and *in vivo*. Gene reporter assays (e.g. luciferase assay) allow to identify specific miRNAs which bind differentially to the wild-type and variant 3’UTRs. Depending on whether or not the miRNA of interest is expressed in the cell line used in luciferase assay, miRNA mimics and/or inhibitors need to be used in the assay to ensure that the observed effect can be attributed to the specific miRNA. mRNA and protein expression should be also compared between different genotypes *in vivo* in relevant samples, taking into account levels of specific miRNAs in individual samples. Finally, based on the known functions of the protein encoded by the gene bearing the miRSNP, appropriate tests should be performed to unravel functional consequences of the miRSNP, which could explain their impact on the disease risk or prognosis. In case no significant effects are observed, an alternative possibility worth considering is an indirect impact of a miRSNP on expression of other genes targeted by the same miRNA through changes in the pool of miRNA available for binding in the cell. It has been proposed that different classes of RNA transcripts – mRNAs, pseudogenes and long non-coding RNAs – compete for miRNA binding, intertwined in a large regulatory network. The “competing endogenous RNA” (ceRNA) hypothesis assumes that RNAs communicate with each other through miRNAs and miRNA recognition sequences [23]. Thus, the effect of a miRSNP may extend beyond expression of the gene bearing the polymorphism, and influence other RNAs regulated by the same miRNA.

In conclusion, polymorphisms in miRNA-binding sites not only offer a possibility of novel diagnostic and prognostic markers in hematological malignancies, but can also help to understand complex regulatory networks of miRNAs in health and disease. With the development of more sophisticated bioinformatics algorithms and accumulation of data from sequencing of cancer genomes, identification of miRSNPs of clinical utility and functional relevance for hematological malignancies should be facilitated.

## Abbreviations

ALL: Acute lymphoblastic leukemia
AML: Acute myeloid leukemia
CDS: Coding sequence
CML: Chronic myeloid leukemia
ceRNA: Competing endogenous RNA
DFS: Disease-free survival
DLBCL: Diffuse large B-cell lymphoma
HL: Hodgkin lymphoma
miRNA: microRNA
miRSNP: miRNA-binding site polymorphism
MM: multiple myeloma
NHL: Non-Hodgkin lymphoma
OR (95% CI): Odds ratio (95% confidence interval)
OS: Overall survival
PFS: Progression-free survival
RFS: Relapse-free survival
SNP: Single nucleotide polymorphism
UTR: 3’ untranslated region
